# Design and Performance Analysis of Robotic Vertebral-Disc Unit with Cable-Driven Mechanism

**DOI:** 10.3390/biomimetics9090512

**Published:** 2024-08-25

**Authors:** Wenshuo Gao, Zhiwei Tian, Famin Duan, Chunxiao Han

**Affiliations:** 1 Laboratory of Robot Mechatronics, University of Rome Tor Vergata, 00133 Rome, Italy; wenshuo.gao@students.uniroma2.eu; 2 Institute of Urban Agriculture, Chinese Academy of Agricultural Sciences, Chengdu 610213, China; tianzhiwei@caas.cn; 3 SDIC Xinjiang Luobupo Postash Co., Ltd., Hami 839099, China

**Keywords:** human spine anatomy, one-vertebral-disc unit, cable-driven mechanism, human-like motions, robotics

## Abstract

The humanoid torso is crucial for the overall performance of a humanoid robot. Developing an effective humanoid spine is essential for enhancing this mechanism. This paper introduces a one-vertebral-disc unit inspired by human spine anatomy. A prototype was created using 3D-printed parts and commercially available components. Two general human-like motions are achieved using two servo motors and two pulleys, reducing the number of servo motors needed. The results indicate that a one-vertebral-disc unit can bend up to 15 degrees. The proposed mechanism functions effectively and successfully mimics human movements. It holds potential for integration into humanoid torsos, enabling efficient performance in human-like tasks in the future.

## 1. Introduction

Humanoid robots are increasingly applied in our lives, and better working performance is also required. A humanoid torso is the key structural part of the body, supporting all other parts of a humanoid robot [1,2]. In general, humanoid robots are characterized by rigid torsos. In terms of improving the performance of completing human-like motions, a torso with a flexible structure can make a difference. According to the structures of the robots developed over these years, a humanoid torso with a humanoid spine structure can provide proper flexibility for a humanoid robot in completing human-like tasks.

Developing humanoid spine mechanisms for applying them in humanoid torsos has become the focus of academic research. In general, a proper mechanism has been successfully developed according to the anatomy of the human spine to eliminate pain caused by bone diseases. Peneie et al. [3] proposed a mechanical spine model with a gear mechanism, consisting of three equal segments. It can complete the targeted human-like motion. However, the structure of the mechanism is complicated. Meanwhile, the gear mechanism requires higher assembling accuracy. The designed spine is manufactured using metal, which increases the weight of the whole mechanism. Therefore, some scholars applied the simplified structures to design and analyze the humanoid spine. Ramalho et al. [4] proposed a robotic spine with a limited number of movable joints based on a proper design strategy as well as by applying measured human spinal motion data. Yu et al. [5] developed a robotic spine composed of parallelogram actuation modules that can complete natural body postures. Ciszkiewicz et al. [6] designed a functional spinal unit and spine as a rigid mechanism by using a constraint equation method. Kuehn et al. [7] developed an artificial spine by combining parallel kinematic mechanisms to improve the effective capabilities of the walking robot’s body.

In these studies, researchers primarily employed a linkage mechanism to achieve rigid body gestures. This approach can increase the degrees of freedom for the humanoid torso in a robot. However, most of these mechanisms were constructed using metal materials, which are not sufficiently lightweight. In addition, using the rigid linkage mechanism makes it lack proper flexibility. Singh et al. [8] developed a human spine module with a cylindrical part and rotatory part as well as a ball inside. The structure is simple and lightweight. It can efficiently execute bending and rotational movements within the designed degrees of freedom while minimizing complexity. However, a proper driving mechanism has to be applied in the mechanism to ensure the proposed spine design works properly.

Some scholars applied different methods and mechanisms to develop a humanoid spine to achieve better working performance in human-like tasks. Ku et al. [9] developed a biological spine actuated by distributed six-link two-dimensional electromechanical actuators. The proposed design can accomplish three distinct motions: left swing, right swing, and S-shape motion. Yet, during the movement of the biological spine, the mechanism may experience increased power consumption. Li et al. [10] proposed a bioinspired humanoid torso with twist angles, actuated by disc-type motors. It can complete human-like motions. Reinecke et al. [11] developed a humanoid torso with a continuum mechanism based on silicone and tendons for actuation, but it has only one unit and has not been applied on a humanoid robot to evaluate its working performance. Wang et al. [12] designed and simplified a flexible robotic spine actuated by shape memory alloy to achieve both bending motion and impact absorption, allowing robots to achieve a variety of postures. It consists of several modules, and flexible tubes are applied as intervertebral discs. Nevertheless, the flexible tubes could influence the stiffness of the whole spine. Kakehashi et al. [13] developed a three-degree-of-freedom continuum spine mechanism for a humanoid robot, which can mimic the structure and motion of the human spine stably. CF rods, pull springs, and push springs are designed with discs of different sizes so that the humanoid spine can move properly. It has proper stiffness. However, the structure is a little complicated and it requires a highly controlled strategy, so users could have problems operating this humanoid spine or even the humanoid robot.

**Figure 1:**
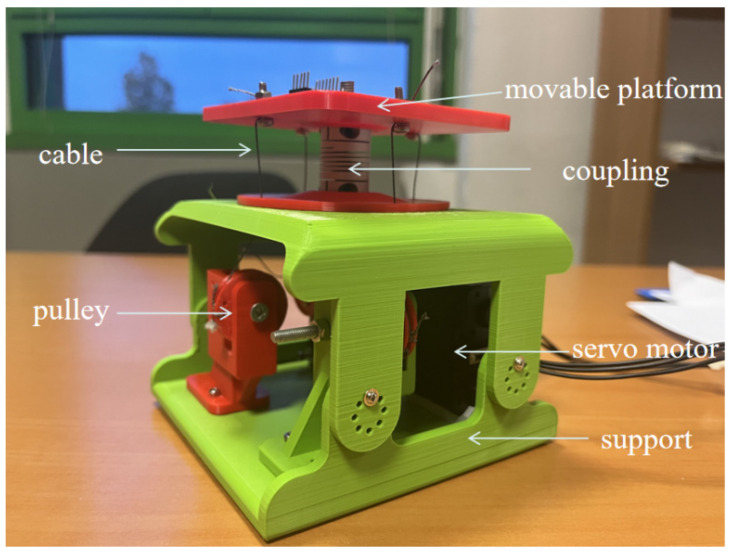
First version of the one-vertebral-disc unit mechanism.

These years, scholars in the Laboratory of Robotics and Mechatronics (LARM2) are also working on humanoid robots as well as a humanoid torso [14]. For example, Cafolla et al. [15] developed the LARMbot torso V1 with three degrees of freedom (DoFs), which is manufactured using 3D-printed parts and market components, actuating by a cable-driven mechanism with four servo motors. Russo et al. [16] conducted a kinematic and dynamic analysis of the proposed structure to evaluate the characterization of the LARMbot torso V1. In addition, a full rotation experiment was conducted to obtain some valuable parameters of the LARMbot torso V1 [17]. In addition, two conceptual vertebral-disc unit designs are proposed and explained for improving the mobility performance of the LARMbot torso V1. Therefore, the one-vertebral-disc unit must be redesigned to develop a new LARMbot torso with better working performance. Based on the conceptual designs, a vertebral-disc unit prototype (regarded as version one) is manufactured, and the characterization is evaluated based on the measured parameters from previous experiments [18], as shown in Figure 1. It can mimic the motion of the human spine with satisfactory performance, addressing some issues found in other designs, such as flexibility and actuation. However, the cables are secured using screws and nuts at the movable vertebral platform, which does not ensure sufficient tension. Additionally, the helical coupling is overly flexible, resulting in stiffness insufficient for supporting the shoulder.

To address the issues identified in the initial version of the one-vertebral-disc unit, new connectors and a novel type of coupling have been implemented. Developing a functional one-vertebral-disc unit is essential for creating a humanoid torso with optimal operational capabilities. The primary objectives of this paper can be summarized as follows:

A. Improvements. According to the previous version of the one-vertebral-disc unit, a new version with proper characterizations has to be developed to improve the working performance. The new mechanism is capable of achieving the stability and flexibility requisites, thereby facilitating the manufacture of a humanoid torso.
B. Mechanism. Four potential driving mechanisms are put forth for consideration. A single option is then selected for manufacture as a prototype. Experimentation serves to verify the possibilities and working performance of the proposed one-vertebral-disc unit mechanism.
C. Motions. In order to develop a humanoid torso, it is essential that the basic unit is capable of performing human-like motions in principle. Consequently, the objective is to design appropriate experiments to verify the functionality of the one-vertebral-disc unit in performing the desired human-like motions.

In this paper, a new one-vertebral-disc unit is proposed and optimized as part of a humanoid torso, inspired by human torso anatomy. A prototype was created using 3D-printed parts and commercially available components. Sensors were strategically installed to measure parameters for evaluating the prototype’s performance. Two types of human-like motions were tested in the experiments. The results were analyzed to characterize the mechanism’s performance in these human-like motion experiments, paving the way for the development of an improved LARMbot torso in the future. The principal contributions of this work can be summarized as follows:

A. A cable-driven mechanism comprising two servo motors: Two servo motors are employed to drive the four cables. In comparison to the use of four servo motors, the number of servo motors is reduced, which may be regarded as a reduction in cost.
B. Proper Properties: The one-vertebral-disc unit is capable of replicating the human-like motions of bending forward and backward, as well as bending left and right.
C. Improvements: The cable-driven mechanism has been enhanced from three cables to two cables in a single loop. A novel coupling has been incorporated to enhance stability and flexibility.

## 2. Biomimetics Principles Spine Anatomy for Human

The human spine plays a pivotal role in the body, serving as a vital structural support system, protecting the spinal cord, facilitating flexibility and movement, absorbing shock, and providing attachment points for muscles and ligaments [19,20]. Studying the human spine offers invaluable insights for designing humanoid torsos that are flexible, stable, and capable of complex movements [21]. The human spine can be conceptualized as comprising multiple units with a similar structural composition. Therefore, it is crucial to focus on a one-vertebral-disc unit before developing a humanoid torso comprising multiple units. Once a single unit is fully functional, it can serve as a model for the entire structure. By analyzing the anatomy of the human spine, it is possible to determine the optimal number of units for a humanoid torso.

**Figure 2:**
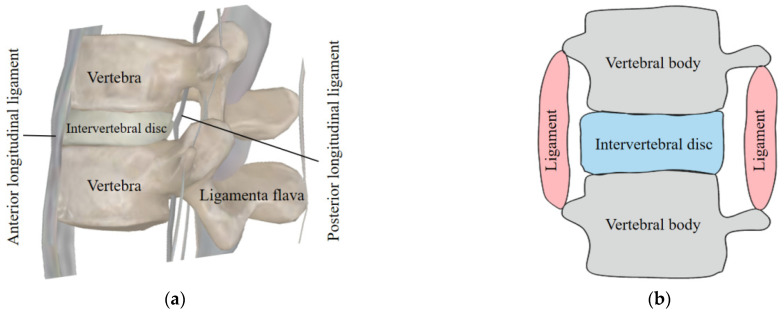
Human anatomy of a human spine: (**a**) human spine; (**b**) mechanical model.

A human spine consists of several units. As shown in Figure 2a, one unit of the human spine is made of a vertebra, intervertebral disc, ligament flava, anterior longitudinal ligament, and posterior longitudinal ligament [22,23]. Based on this anatomy, a mechanical model is proposed as shown in Figure 2b, including vertebral bodies, intervertebral discs, and ligaments. The vertebra body can be regarded as moving through the pulling motion from the ligament. The intervertebral disc is made of flexible material. It combines two vertebra bodies and can decrease external impacts. The whole human torso is made of several of these units stacked on top of each other in a serial kinematic chain.

## 3. Conceptual Designs

**Figure 3:**
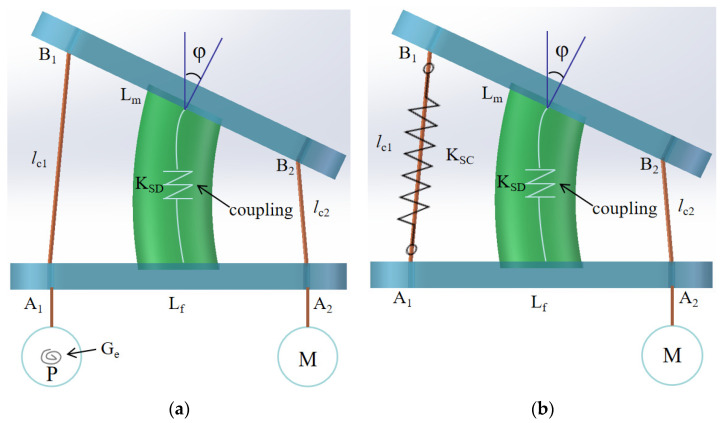
Conceptual designs for a new vertebral-disc unit: (**a**) with torsion spring; (**b**) with tension spring.

As shown in Figure 3, two designs with springs are proposed. Figure 3a shows a design with a torsion spring, which consists of two vertebral platforms, two cables, a coupling, a pulley with a torsion spring, and a servo motor. A_1_A_2_ is the fixed vertebral platform with length L_f_. The cables with length *l*_c1_ and *l*_c2_ go through the fixed vertebral platform and connect with pulley G_c_ and servo motor, respectively. The movable vertebral platform B_1_B_2_ with length L_m_ can perform the motion as actuated by the cooperation of the servo motor and the pulley with the torsion spring. The *φ* angle of the movable vertebral platform shows the relative motion between the two vertebral platforms. The tension forces on the cables are indicated as T_c1_ and T_c2_. The one-vertebral-disc unit can perform motion in several steps. For instance, when the servo motor rotates 90 degrees, the unit is in the home position. At 0 degrees, the moving vertebra bends appropriately to the right. At 180 degrees, the movable vertebral platform bends to the left at a suitable angle. During this process, the pulley with the torsion spring rotates accordingly. The primary design consideration involves the proper design of the torsional spring.

The other one is a design with a tension spring, which is made of two vertebral platforms, a servomotor, a coupling, and a tension spring, as shown in Figure 3b. A cable goes through the fixed vertebral platform A_1_A_2_ with length L_f_. The length of the cable is *l*_c_ with tension force T_c_. The tension spring is characterized by length *l*_SC_ and a coupling coefficient K_SC_, and its tension on it is indicated as Ts given by T_c1_. The servo motor rotates to adjust the length of the cable and, consequently, the length of the spring. The force and length of the tension spring change in coordination. The upper vertebral platform moves at an appropriate angle relative to the fixed platform. During this process, the servo motor operates similarly to the one in the torsion spring design, causing the movable vertebral platform to bend in a comparable manner [17].

**Figure 4:**
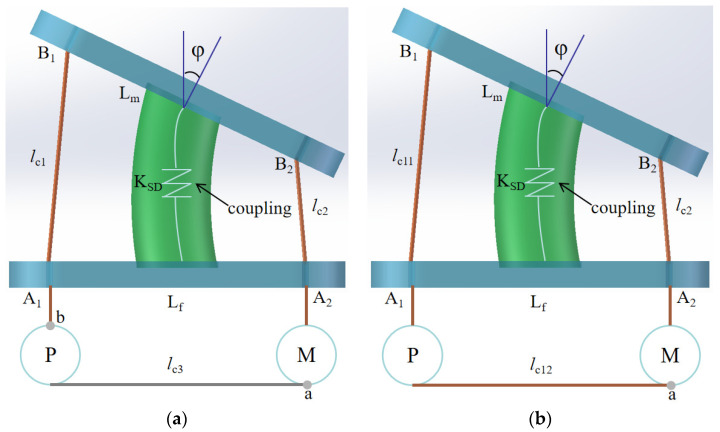
Conceptual designs for a new vertebral-disc unit: (**a**) with three cables [18]; (**b**) with two cables.

In addition, another two designs with pulley are proposed, as shown in Figure 4. In Figure 4a, it is a design with a pulley and three cables. It consists of a movable vertebral platform, a fixed vertebral platform, a coupling, a pulley, a servo motor, and three cables. The L_f_ is the length of fixed vertebral platform A_1_A_2_. Cable 1, with length *l*_c1_, goes through A_1_ and is knotted on pulley P at point a. Cable 2, with length *l*_c2_, goes through A_2_ and is knotted on servo motor M at point b. Cable 3, with length *l*_c3_, is knotted from b to a, which is used to transfer the torque from servo motor M to pulley P. The movable vertebral platform B_1_B_2_, with length L_m_, can perform the motion as actuated by the servo motor and the pulley working together. The *φ* angle of the movable vertebral platform shows the relative motion between the two vertebral platforms. The tension forces on the cables are indicated as T_c1_ and T_c2_. The one-vertebral-disc unit can perform motion in several steps. For instance, when the servo motor rotates 90 degrees, the unit is in the home position. At 0 degrees, the movable vertebral platform bends appropriately to the right. At 180 degrees, the moving vertebra bends to the left at a suitable angle. During this process, the pulley rotates at the same angles, connected by cable 3.

Another design with a pulley and two cables is proposed to realize the same motion of the one-vertebral-disc unit, as shown in Figure 4b. It consists of a movable vertebral platform, a fixed vertebral platform, a coupling, a pulley, a servo motor, and two cables. The vertebral platform A_1_A_2_ with a length of L_f_ is fixed on the support. Cable 1 goes through A_1_ and pulley P and is knotted on the servo motor at point a. *l*_c11_ is the length of cable 1 between A_1_ and B_1_. *l*_c12_ is the length of cable 1 between A_1_ and point a. Cable 2 goes through A_2_ and is knotted at the same point a. *l*_c2_ is the length of cable 2. The function of pulley P is to keep cable 1 in the proper direction and transfer the torque from servo motor M. The movable vertebral platform B_1_B_2_ with length Lm can perform the motion as actuated by the servo motor and the pulley working together. The *φ* angle of the movable vertebral platform shows the relative motion between the two vertebral platforms. The mechanism can complete the same motions through a similar motion from the servo motor.

In conclusion, these four designs share several similarities. Firstly, they are cable-driven mechanisms, akin to the structure of the human spine. Additionally, they effectively perform basic motions, such as bending left and right. Moreover, each design utilizes only one servo motor, reducing the number of servo motors required in the mechanism and potentially lowering the cost of manufacturing a prototype in the future. However, there are also notable differences among these designs. In the designs shown in Figure 3, springs are used to store and release tension, working in conjunction with a servo motor to rotate the movable vertebral platform to the desired angle. The spring parameters must be accurately calculated to ensure stable motion. Conversely, the designs in Figure 4 incorporate pulleys. The pulleys guide the cables in the correct direction and successfully transfer torque from the servo motor. As the servo motor rotates, the effective length of the cables changes uniformly. When the distance A_1_B_1_ between two vertebral platforms decreases by value *e*, distance A_2_B_2_ between the two vertebral platforms increases by approximately *e* simultaneously.

## 4. One-Vertebral-Disc Unit Design

**Figure 5:**
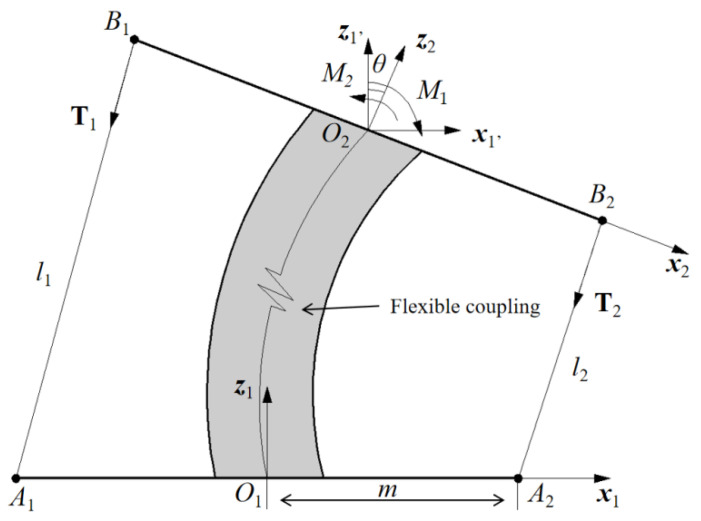
Kinematic analysis of the proposed one-vertebral-disc unit mechanism.

It is important to figure out the relationship between the fixed vertebral platform and the movable vertebral platform and obtain the position of the movable vertebral platform. Thus, a kinematic analysis is conducted by using the D-H method. The D-H method is a general method for expressing the position of the end effector relative to the base coordinate system, finally being deduced by sequential transformation, so as to establish the kinematic equation of the mechanism. The D-H method is applied on the proposed one-vertebral-disc unit mechanism. A kinematic analysis is conducted as shown in Figure 5. A_1_A_2_ is shown as the fixed vertebral-disc platform. B_1_B_2_ is shown as the movable vertebral-disc platform. These vertebra platforms are designed for mimicking the vertebra unit of the human spine. A_1_B_1_ and A_2_B_2_ are two cables that are actuated by servo motors. The length of the cables A_1_B_1_ and A_2_B_2_ are indicated as *l*_1_ and *l*_2_, respectively. In addition, the tension forces on the cables are indicated as T_1_ and T_2_, respectively. The flexible coupling is used for realizing the function of the intervertebral disc. Two Cartesian coordinate systems are assumed on the fixed vertebra and moving vertebra as O_1_*x*_1_*y*_1_*z*_1_ and O_2_*x*_2_*y*_2_*z*_2_, respectively. O_1_ and O_2_ are at the center of the vertebrae. The model does not have motion in the direction Y, so the analysis is conducted on the O*xz* plane only. The rotated angle of the moving vertebra B_1_B_2_ is indicated by *θ* deg, which is changed by rotating around the Y axis. The length of O_1_A_2_ is indicated as *m*. During the moving process, the generated torques on the movable vertebral-disc platform are indicated as M_1_ and M_2_. *l* is the distance of O_1_O_2_ [17].

To express the position of the movable vertebral-disc platform, some parameters are applied in the model, as follows:

*a*_i_: the distance traveled from *z*_i_ to *z*_i+1_ along the *x*_i_ axis;

*α*_i_: the angle of rotated from *z*_i_ to *z*_i+1_ along the *x*_i_ axis;

*d*_i_: the distance traveled from *x*_i−1_ to *x*_i_ along the *z*_i_ axis;

*θ*_i_: the angle rotated from *x*_i−1_ to *x*_i_ along the *z*_i_ axis.

The moving process can be expressed as homogeneous transformation matrix T, as follows [17,18]:

(1) Tii−1=RX(αi−1)×DX(αi−1)×RZ(θi)×DZ(di)

Then, the transformation matrix T can be expressed as

(2) Tii−1=[cosθi−sinθi0ai−1sinθicosαi−1cosθicosαi−1−sinαi−1−sinαi−1disinθisinαi−1cosθisinαi−1cosαi−1cosαi−1di0001]

Based on the motion of the movable vertebral-disc platform, the end position of B_1_B_2_ can be expressed by matrix T:

(3) T10=[cosθ−sinθ00sinθcosθ00001l0001]

A human torso can perform three types of motions. Consequently, a vertebral-disc unit must have three degrees of freedom (DoFs): flexion–extension, lateral bending, and transverse rotation, which correspond to bending forward and backward, bending left and right, and rotational motion, respectively. Generally, the motions of bending forward and backward and bending left and right are the two basic movements frequently utilized in human-like motion for a humanoid robot. Therefore, in the design of a vertebral-disc unit, only these two basic motions are considered: bending forward and backward and bending left and right.

**Figure 6:**
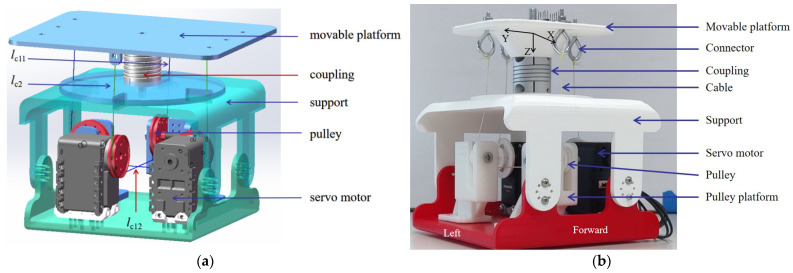
One-vertebral-disc unit mechanism: (**a**) CAD model; (**b**) prototype.

To achieve the two basic motions, two groups of the design mentioned in Figure 4b are combined. The CAD model is shown in Figure 6a. The one-vertebral-disc unit consists of two pulleys, two servo motors, four cables, two platforms, a coupling, and support. The flexible coupling is of appropriate flexibility and stiffness. Additionally, it exhibits a structural resemblance to a spring, which enables it to dissipate some shock.

Based on the design shown in Figure 6a, the prototype is manufactured by using 3D-printed parts and market components. As shown in Figure 6b, the one-vertebral-disc unit consists of pulleys, servo motors, a coupling, cables, a movable platform, and supports. The radius of the pulleys is 6 mm. The type of coupling is winding flexible CNC coupling. The maximum torque is 3 N·m. The admissible angular misalignment is 0.5 deg. The material is Al, chromed. The weight is 50 g. The length and diameters are 25 cm and 30 cm, respectively. An eye screw is applied to adjust the distance so that the cables can be tightened enough and have proper tension force. Force sensors are applied between the movable vertebral platform and the washer, which are used to measure the tension force on cables. A cable is knotted at the ring of the eye screw. The eye screw is screwed with a nut. A washer is applied to increase the surface area between the nut and the movable vertebral platform so that the force sensors can be installed in a suitable space.

## 5. Experimental Set-Up

**Figure 7:**
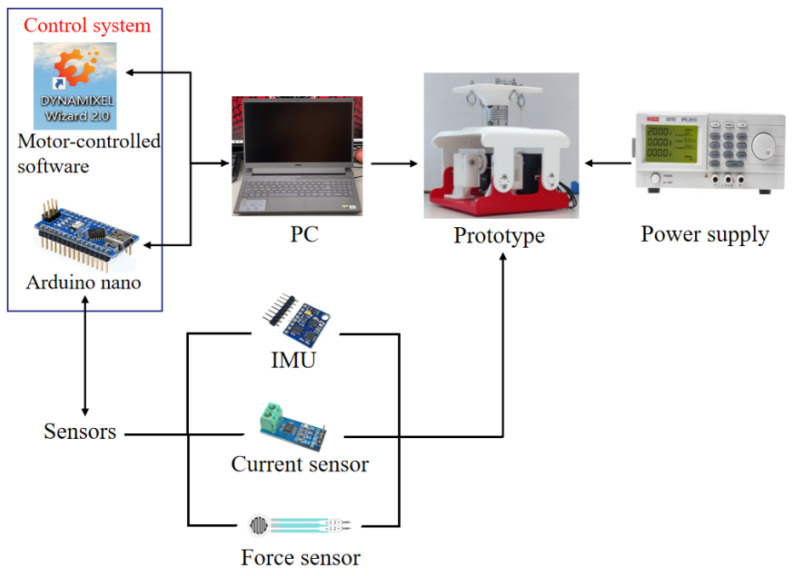
Control system of the experiments.

As shown in Figure 7, the experimental set-up consists of the prototype in Figure 6b, a control system, a PC, a power supply, and sensors. The control system is made of motor controllers, an Arduino nano board, an IMU, a current sensor, and force sensors for measuring motion parameters, power consumption, and tension forces on the cables, respectively. Force sensors (type: FSR 400) are applied to measure the force between the cables and movable platform, which can be regarded as tension force. The force sensitivity range is ~0.2 N–20 N, which is enough for measuring the forces in experiments with proper accuracy. There are two kinds of control strategies that can be applied to control the mechanism, including an open-loop control strategy and a closed-loop control strategy. To explore the maximum bending angle of the one-vertebral-disc unit, an open-loop control strategy is applied. Because of the servo motors (type: AX-12A), the software DYNAMIXEL Wizard 2.0 is applied to actuate the servo motors directly. It provides an easy-control method for the whole system in Figure 7. Some sensors will measure the parameters during the testing process. However, a closed-loop control strategy has to be developed for the humanoid torso in the future, so that the bending angles can be controlled stably and accurately.

**Figure 8:**
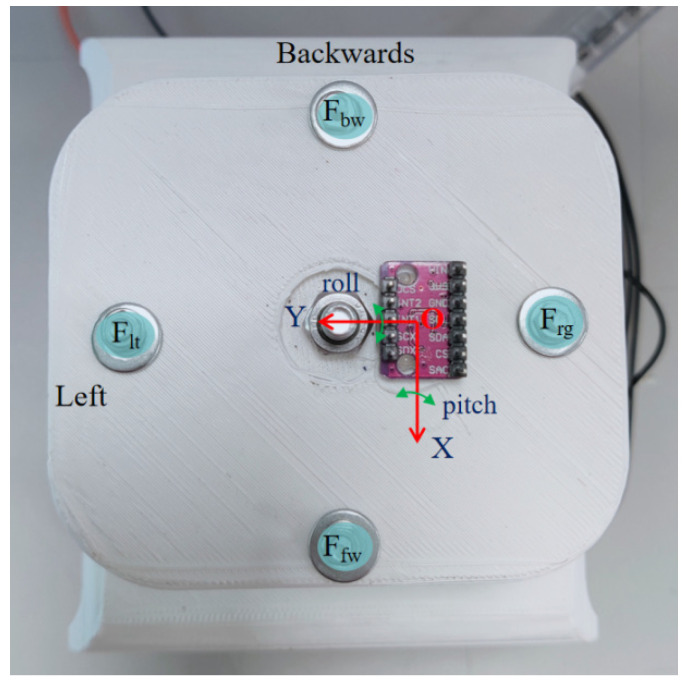
Indications of the force sensors and IMU sensor on the movable platform.

Figure 8 shows the IMU sensor and force sensor reference frames. According to the results of the pre-experiments, the one-vertebral-disc unit can bend around a maximum of 18 deg. If the target bending angle of the one-vertebral-disc unit is 18 deg, losing tension on the cable happens easily at the other side (with the pulley only). In addition, the greater the bending angle obtained, the more tension force on the cables is required. Thus, the target motions of the experiments are bending right and left and bending forward and backward, which are two basic human-like motions with numerical targeted values of around ±15 deg. Two experiments are proposed to evaluate the characterizations of the new one-vertebral-disc unit prototype, as follows:

Mode 1: Bending left and right. The vertebral disc bends left at first (servomotor −90 deg from 0 deg), then right (servomotor −90 deg to +90 deg), and finally moves back to the original position.

Mode 2: Bending forward and backward. The vertebral disc bends forward at first (servomotor −90 deg from 0 deg), then backward, (servomotor −90 deg to +90 deg), and finally moves back to the original position.

## 6. Results

**Figure 9:**
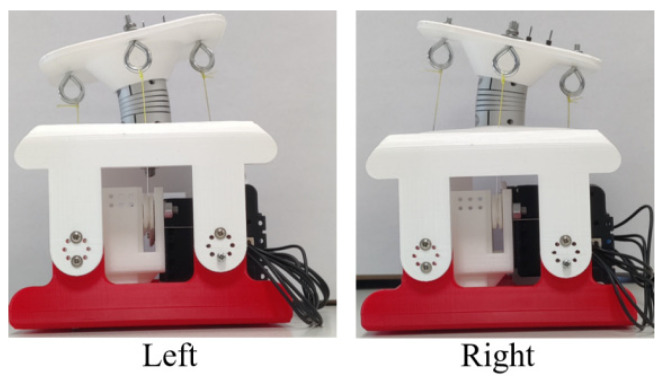
Motion results of one-vertebral-disc unit prototype in mode 1.

**Figure 10:**
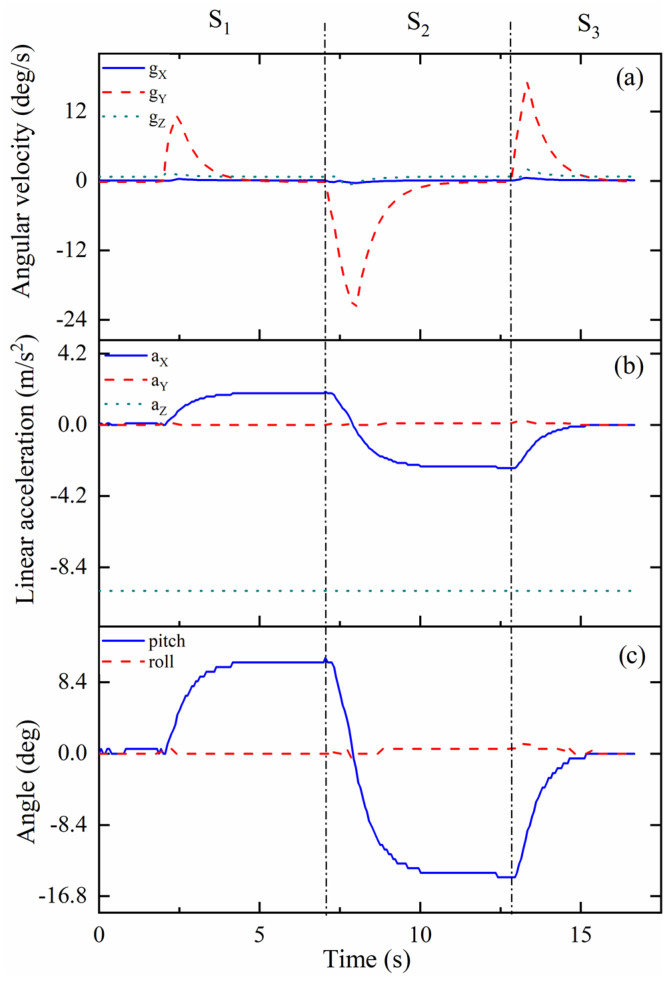
Kinematic results of one-vertebral-disc unit in mode 1: (**a**) angular velocity; (**b**) linear acceleration; (**c**) angle.

**Figure 11:**
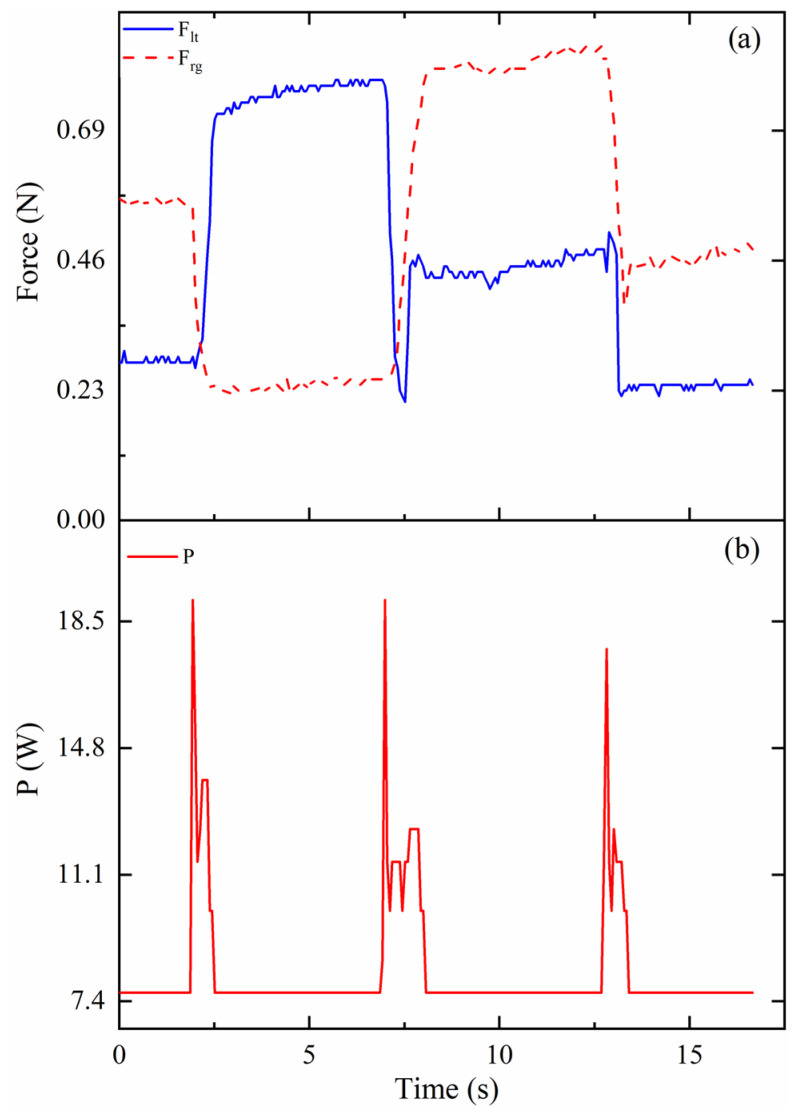
Results of the tension force on cables and consumed power in mode 1: (**a**) Force (N); (**b**) P (W).

**Figure 12:**
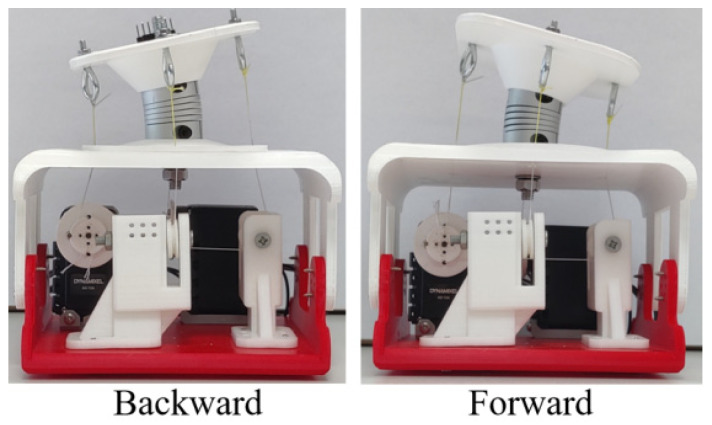
Motion results of one-vertebral-disc unit prototype in mode 2.

**Figure 13:**
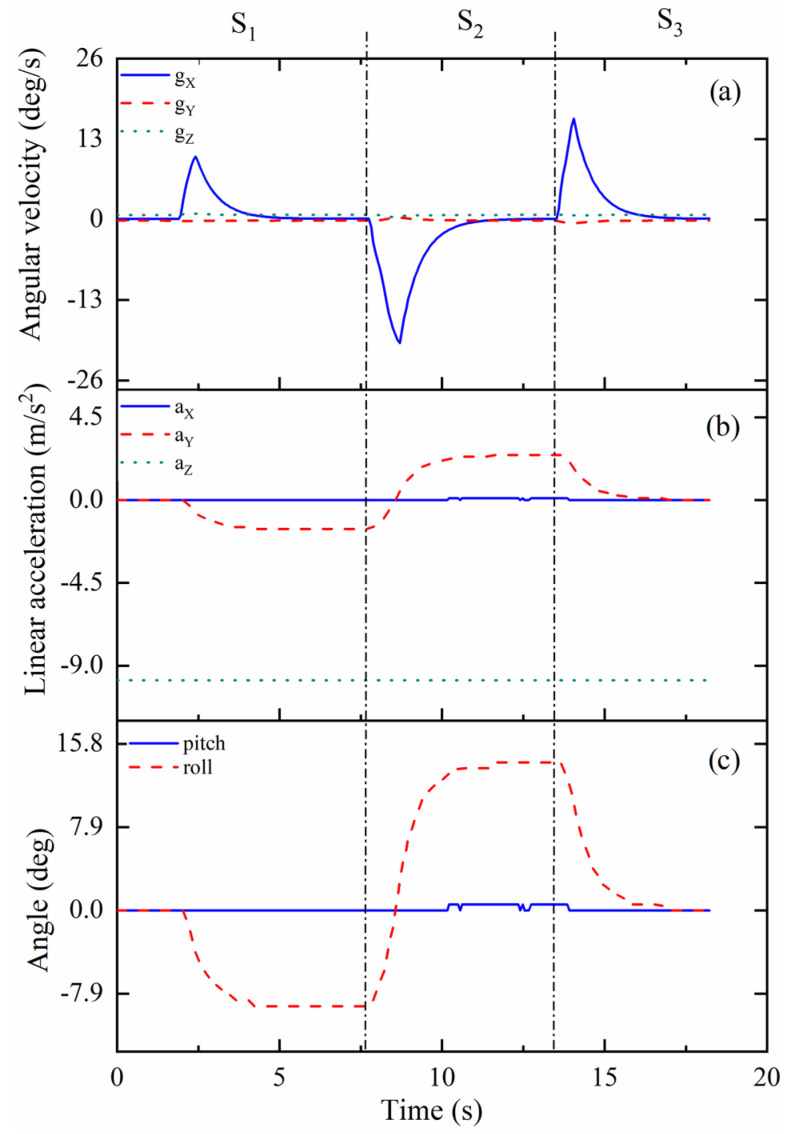
Kinematic results of one-vertebral-disc unit in mode 2: (**a**) angular velocity; (**b**) linear acceleration; (**c**) angle.

**Figure 14:**
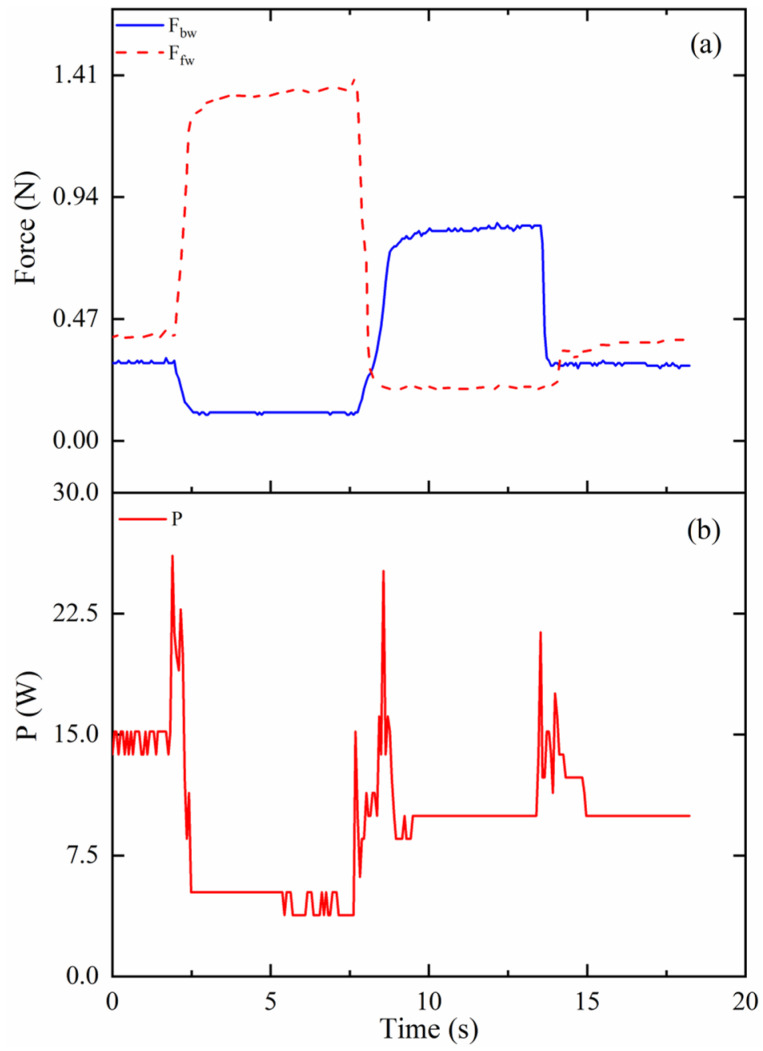
Results of the tension force on cables and consumed power in mode 2: (**a**) Force (N); (**b**) P (W).

The results are obtained after conducting the experiments proposed in two modes, as shown from Figure 9, Figure 10, Figure 11, Figure 12, Figure 13 and Figure 14.

Mode 1: motion in left and right directions.

In mode 1, the experiment is conducted as planned. The mechanism runs normally, and the prototype moves as expected. Motion snapshots are shown in Figure 9.

The kinematic characterization of the one-vertebral-disc unit prototype in mode 1 is presented as in Figure 10. The whole experimental process can be divided into three steps. At the beginning, the prototype bends left, which is regarded as the step 1 in this process, indicated as S_1_. The motion S_1_ starts from around 2 s and ends at around 4 s. As shown in Figure 9a, the maximum angular velocity can reach 12 deg/s in direction Y when the time is around 2.5 s. The angular velocities in directions X and Z are near 0 deg/s until the end of the whole moving process in mode 1. When the time is around at 4 s, the linear acceleration in direction X reaches to the maximum, around 2 m/s^2^. In terms of the values in other directions, the linear accelerations in directions Y and Z are at stable levels until the end of the experiment in mode 1, around 0 m/s^2^ and 9.8 m/s^2^, respectively. At the same time, the bending angle in pitch of the prototype can reach around 11 deg, regarded as the bending angle in left. As for the step 2 in mode 1, the prototype moves from the left to right directly. The motion starts from around at 7 s, and ends around at 12 s. When the time is around 8 s, the value of the angular velocity in direction Y reaches to around −24 deg/s. However, when the time is around at 12 s, the linear acceleration in opposite direction X reaches to maximum, around 2 m/s^2^. The bending angle in pitch is 15 deg, regarded as the bending angle in right. When the time is 13 s, the prototype moves back to the original position from the right, indicated as the step 3 in mode 1.

It is also important to obtain the tension force on the cables so that a better evaluation can be made on the working performance of the one-vertebral-disc unit prototype. Results of the tension force on the cables in mode 1 are shown in Figure 11a. When the time is around at 2 s, the cable on the left pulls the movable vertebral platform, and the prototype bends left. The tension force on the left cable, indicated as F_lt_, increases, reaching to around 0.75 N. However, the tension force on the right decreases to around 0.22 N from around 0.56 N. When the prototype bends from left to right, the tension force on the cables changed. The tension force on the left and right are around 0.45 N and around 0.8 N, respectively. During the whole process of the experiment in mode 1, the maximum required power is 19 W, as shown in Figure 11b.

Mode 2: motion in forward and backward direction.

As for the mode 2, the experiment is conducted as planned. The snapshot results of the experiment in mode 2 are shown in Figure 12. The prototype works as expected, bending forward and backward.

Kinematic results in mode 2 are shown in Figure 13. The moving process is divided into three steps, indicated as S_1_, S_2_, and S_3_. As for the step S_1_, the motion process starts from around 2 s, and ends around 4.5 s. As shown in Figure 13a, the maximum of angular velocity is 10 deg/s in direction X. In terms of the angular velocity in directions Y and Z, the values remain stable until the end of the whole experiment in mode 2, near 0 deg/s. As for the linear acceleration in Figure 13b, it reaches its maximum, −2 m/s^2^, in direction Y. The values of the linear acceleration in directions X and Z remain stable until the experiment in mode 2 is complete; these values are 0 m/s^2^ and −10 m/s^2^, respectively. In step S_1_, the prototype bends forward and can reach 10 deg in roll, as shown in Figure 13c. In addition, during the step S_2_ process, the movement of the prototype starts from 7.5 s, and ends around 12 s. The angular velocity in direction X can reach −20 deg/s. The linear acceleration in direction Y changes from −2 m/s^2^ to 2 m/s^2^. Meanwhile, the bending angle changes from 10 deg forward to 15 deg backward in roll. In step S_3_, the prototype bends from backward to the original position, moving from 13.5 s and ending at 16 s. The angular velocity in direction X can reach 15 deg/s. The linear acceleration in direction Y changes from 2 m/s^2^ to 0 m/s^2^. As for the bending angle, it changes from 15 deg to 0 deg in roll.

As shown in Figure 14a, the tension force on the cables in mode 2 are measured, and the results are plotted. When the prototype bends forward, as S_1_ in mode 2, the tension force on the forward cable, F_fw_, increases to 1.35 N from 0.4 N. However, the tension force on the backward cable, F_bw_, decreases to 0.1 N from 0.3 N. When the prototype completes the S_2_ of the experiment in mode 2, the tension force on the forward cable decreases from 1.35 N to 0.2 N. At the same time, the tension force on the backward cable increases from 0.1 N to 0.8 N. Finally, the prototype moves to the original position, and the values of the tension force on the cables change to the same as those at the beginning. During the whole experiment process in mode 2, the required maximum power is around 26 W, as shown in Figure 14b.

Compared with the previous version reported in [18], the improved one-vertebral-disc unit addresses several issues. The updated version features a different type of coupling, enhancing the mechanism’s supporting functions. Additionally, appropriate connectors are used between the movable vertebral-disc platform and cables, allowing for easier measurement of cable tension. A more powerful servo motor is also incorporated, capable of driving a humanoid torso with multiple units. However, this requires significantly more power than the previous design. The improved unit achieves smaller bending angles due to the different parameters of the new couplings. The new prototype offers proper stability and flexibility. Furthermore, the improved design facilitates the combination of several vertebral-disc units into a humanoid torso with optimal performance. Compared to other designs, the developed one-vertebral-disc unit provides an appropriate balance of rigidity and flexibility, functioning as a modular component with combinatorial flexibility. By combining a specific number of units, the desired structure can be achieved. The design is more adaptable and avoids structural and performance singularities through the use of couplings. However, the measured parameters are limited to general metrics for evaluating the motion performance of the one-vertebral-disc unit, which are sufficient for providing references for the development of a humanoid torso. In the future, additional parameters, such as the relationship between tension force and required power, should be measured and calculated to further evaluate the working performance of the humanoid torso.

In this paper, we proposed four conceptual designs of the vertebral-disc unit mechanism according to the anatomy of a human spine unit. Based on an acceptable and convenient-design mechanism of the one-vertebral-disc unit, a prototype was manufactured by using 3D-printed parts and market components. Experiments in two modes were conducted to evaluate the performance of the whole prototype. The proposed one-vertebral-disc unit mechanism could complete the desired human-like motion: bending left and right and bending forward and backward. Consistent with previous studies, the proposed design of the one-vertebral-disc unit is with proper kinematic characterization and has lower power requirements. The proposed mechanism can work effectively. At the same time, the proposed mechanism can avoid loose tension problems on cables during the moving process in experiments, because all results of the tension force on the cables are over 0 N. In addition, the connectors among the cables, the force sensors, and the movable vertebral platform are flexible so that the measured tension forces are changed, compared with the values at the beginning although the prototype is at the same position. However, the bending angles do not reach the designed value of 15 deg at some specific positions. This is because the applied couplings are not an isotropic structure. In addition, the IMU sensor is installed near the center of the movable vertebral platform, causing errors in the measured results. However, when assembling a prototype with multiple vertebral-disc units, it is crucial to adjust the couplings to maintain proper positions and orientations with reasonable arrangements. This ensures that the structure of the prototype with several vertebral-disc units can be considered as isotropic as possible. In the future, a humanoid torso can be developed for a humanoid robot based on the results of the one-vertebral-disc unit. It is anticipated that a humanoid robot with a developed humanoid torso will be capable of performing general human-like tasks within a home environment, such as grasping a bottle of water. Additionally, the proposed mechanism can be applied to a humanoid torso in the future. Potential applications of the prototype include its use as a movable platform to assist with motion and increase the workspace of manipulators. For example, similar mechanisms could be installed on a wheeled vehicle as a movable platform to assist a humanoid hand in picking vegetables in a greenhouse.

## 7. Conclusions

In this paper, based on the anatomy of the human spine, a prototype with a one-vertebral-disc unit was developed from one of four conceptual designs. Sensors were employed to measure parameters for evaluating the prototype’s performance. The kinematic results indicated that the one-vertebral-disc unit functions effectively, achieving a maximum bend of approximately 15 degrees, making it suitable for various operational conditions. The tension force on the cables was less than 1.5 N during human-like motions. The required power for driving the servo motors was less than 27 W, indicating low power consumption. In the future, a humanoid torso prototype with an appropriate number of vertebral-disc units can be developed to serve as part of a humanoid robot.

## Data Availability

The data used to support the findings of this study are available from the corresponding author upon request.
